# Dissecting stress-activated protein kinase (SAPK)–signaling pathways using multiplex gene knockout HeLa cells

**DOI:** 10.1016/j.jbc.2025.110901

**Published:** 2025-11-04

**Authors:** Chihiro Ito, Mirei Yamamoto, Nozomi Yokota, Nanoka Nakamura, Misaki Shiomi, Misato Kizu, Moe Ichinose, Shinobu Fujii, Toshihiro Fujii, Rikiro Fukunaga

**Affiliations:** Faculty of Pharmacy, Department of Biochemistry, Osaka Medical and Pharmaceutical University, Takatsuki, Osaka, Japan

**Keywords:** JNK, p38 MAPK, CRISPR–Cas, signal transduction, gene expression, SAPK, immediate-early gene, multiplex KO

## Abstract

The stress-activated protein kinase (SAPK) family consists of three c-Jun N-terminal kinase (JNK) and four p38 members. To explore the isotype-specific or overlapping roles of SAPK members, HeLa-derived multiplex SAPK-KO cells, such as JNK1/2/3-triple KO, p38α/β/γ/δ-quadruple KO, and JNK1/2/3/p38α/β/γ/δ-septuple KO cells, were generated using the CRISPR–Cas9 method. Also, “sole survivor” (ss)-hextuple KO cells, in which only one of seven SAPK genes remains intact, were generated. Western blot analyses using phospho-specific antibodies for SAPK substrates showed that serum- or anisomycin-induced phosphorylation of MAPKAPK2, MSK1, Mnk1, and CREB (cyclic AMP response element–binding protein)/ATF-1 largely depended on p38, whereas anisomycin-induced phosphorylation of c-Jun/JunD depended on JNK. Similar analyses using the ss-hextuple KO cell lines revealed that JNK1 rather than JNK2 contributed to the c-Jun/JunD phosphorylation, whereas p38α was the primary species phosphorylating the examined p38 substrates. Expression analyses of three typical immediate-early genes, c-Jun, EGR1, and c-Fos, demonstrated that JNK1 and JNK2 are responsible for c-Jun expression induced by interleukin-1β, tumor necrosis factor-α, UV-C, and heat shock (HS), whereas p38 is predominant in EGR1 expression induced by UV and HS and in c-Fos expression induced by the cytokines, UV, and HS. On the other hand, neither JNK nor p38 contributed significantly to the cytokine-induced EGR1 expression, suggesting complicated SAPK-signaling mechanisms that regulate immediate-early gene expression. Together, these results demonstrate the utility of the comprehensive multigene KO and ss-KO strategy in dissecting intracellular signaling pathways consisting of multiple family members.

Mitogen-activated protein kinase (MAPK) signaling pathways are activated by a range of extracellular signals and are central to intracellular signal transduction leading to gene expression activation and various cellular functions. Extracellular signal-regulated kinase (ERK) is activated downstream of Ras and is pivotal in cellular survival, proliferation, and differentiation, whereas c-Jun N-terminal kinase (JNK) and p38 MAPK are primarily activated by inflammatory cytokines or environmental stresses, including UV light, hyperosmosis, and heat shock (HS), with the latter kinases categorized as the stress-activated protein kinase (SAPK) family (1, 2, 3). The JNK family consists of three members: JNK1, JNK2, and JNK3. The former two kinases are expressed ubiquitously and carry similar and overlapping functions, whereas JNK3 is primarily expressed in neuronal cell lineages with functions distinct from other members (2). For p38 MAPK, there are four family members: p38α, p38β, p38γ, and p38δ. In most tissues, p38α is abundantly expressed and is likely the predominant family species, whereas p38β is less abundantly expressed, followed by p38γ and p38δ, which are expressed in restricted cell types (3, 4, 5).

Traditional gene KO strategies using mouse embryonic stem cells have uncovered physiological functions of SAPK members. JNK1-, JNK2-, or JNK3-single KO mice develop normally, whereas JNK1/2-double KO (DKO) results in embryonic lethality (6, 7, 8, 9, 10, 11, 12, 13). Mice deficient in p38α also exhibit embryonic lethality (14, 15, 16), but p38β-, p38γ-, and p38δ-single KO mice and p38γ/δ-DKO mice are viable and fertile (17, 18). Studies evaluating intracellular signaling pathways using KO-mice–derived embryonic fibroblasts or hematopoietic cells yielded conclusive evidence for the physiological functions of the SAPK family (2, 3, 19). However, for simultaneous multigene studies, the KO mice–based strategy is impractical, as generation, maintenance, and analysis of mice deficient in more than three genes is laborious and time consuming. It is due to this complexity that the detailed understanding of the isotype-specific or overlapping roles shared by the JNK and p38 family in modulating stress or inflammatory responses remains elusive.

CRISPR–Cas9 technology enables multiple gene KOs in most types of cultured cell lines used to study intracellular signal transduction. We recently developed a simple and efficient method to generate simultaneous multigene KO cells from cultured human cell lines (20). With this strategy, at least seven genes can be simultaneously knocked out by simple cotransfection of multiple pX330-derived plasmids, each of which expresses a chimeric sgRNA together with Cas9. An advantage of this cotransfection method is that, once targeting plasmids are established, any combination of multigene KO can be achieved without constructing new multicassette plasmids.

Herein, we generated a series of HeLa-derived multiplex SAPK-KO cells to examine isotype-specific or overlapping roles of kinase family members. Analyses using JNK1/2/3-triple KO (TKO), p38α/β/γ/δ-quadruple KO (QKO), JNK1/2/3/p38α/β/γ/δ-septuple KO (SKO), and the sole-survivor-hextuple KO (ss-HKO) cells clarified both phosphorylation specificities of substrate proteins shared by JNK and p38 as well as the distinct roles in inducing various immediate-early genes, including Jun, Fos, and EGR family members.

## Results

### Generation of SAPK-multiple KO cells and ss-HKO cells

**Figure 1:**
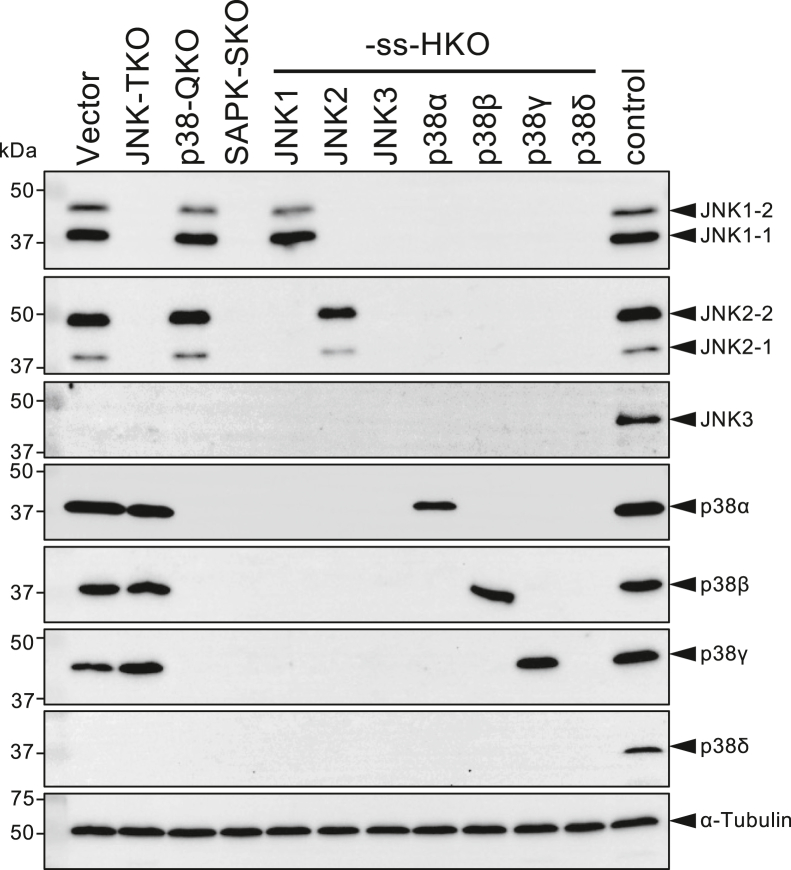
**Generation of HeLa-derived SAPK-MKO cells and sole-survivor HKO cells.** Total cell lysates prepared from HeLa-derived vector control (Vector), JNK-TKO, p38-QKO, SAPK-SKO, and seven ss-HKO cell lines were analyzed by Western blotting using antibodies against JNK1, JNK2, JNK3, p38α, p38β, p38γ, p38δ, and α-tubulin. As positive controls, cell lysates from neuroblastoma SH-SY5Y cells (for JNK3), monocytic leukemia THP1 cells (for p38δ), and HeLa cells (for all the others) were electrophoresed in the right-side lane. HKO, hextuple KO; JNK, c-Jun N-terminal kinase; MKO, multiple KO; QKO, quadruple KO; SAPK, stress-activated protein kinase; SKO, septuple KO; ss, sole survivor; TKO, triple KO.

To generate multigene-KO HeLa cells using the CRISPR–Cas9 system, two pX330-derived targeting plasmids were constructed for each of the JNK1, JNK2, JNK3, p38α, p38β, p38γ, and p38δ genes (Table S1). Although JNK3 and p38δ are known to be expressed only in limited cell lineages and are presumably not expressed in HeLa cells, we performed similar KO operations on these genes, as we were concerned that disrupting all active family genes might induce ectopic expression of the remaining dormant member through a compensatory mechanism. HeLa cells were transfected with each plasmid separately, and KO efficiencies were evaluated by Western blot analyses. Almost all targeting constructs effectively disrupted target genes at the Western blot detection sensitivity (Fig. S1). Then, multiple KO (MKO) cells, including JNK1/2/3-TKO (JNK-TKO), p38α/β/γ/δ-QKO (p38-QKO), and JNK1/2/3/p38α/β/γ/δ-septuple KO (SAPK-SKO) cells, were generated by cotransfection of HeLa cells with mixtures of the selected plasmids shown in Table S1, using a previously reported method (20). In addition, “sole survivor” (ss) KO cells were generated in which only one of the seven SAPK genes remains undisrupted. For example, JNK2/3/p38 α/β/γ/δ-HKO cells were designated as JNK1-ss-HKO cells. Western blot analyses of whole-cell lysates with respective specific antibodies indicated that, like parental HeLa cells, the HeLa-derived control cells (Vector), generated by transfection of the pX330 vector plasmid, expressed JNK1, JNK2, p38α, p38β, and p38γ but not JNK3 or p38δ (Fig. 1). In contrast, no protein products of the genes targeted by the respective mixture of targeting plasmids were detected in JNK-TKO, p38-QKO, and SAPK-SKO cells, indicating efficient multigene disruption, as reported previously (20). Furthermore, all ss-HKO cells expressed only the untargeted gene. Ectopic expression of JNK3 or p38δ was not observed in any of the KO cells. No apparent difference in growth rate or cellular morphology was observed between wild-type HeLa cells and multiplex KO cells, indicating that none of the seven SAPKs are essential for survival or proliferation (data not shown).

To examine whether JNK-TKO or p38-QKO affects the magnitude of stimulation-dependent activation of the respective remaining p38s or JNKs *via* crosstalk or feedback signaling, the activating phosphorylation of JNKs and p38s in the KO cell lines was analyzed. Cell stimulation with fetal calf serum (FCS) strongly activated ERKs and weakly activated p38s, whereas anisomycin treatment activated both JNKs and p38s but not ERKs (Fig. S2). No obvious change in activation magnitude for either the JNK or p38 subfamily was induced by KO of the other subfamily. In contrast, FCS-stimulated ERK activation was slightly enhanced in SAPK-SKO cells.

### p38α and p38β but not JNKs are responsible for stress-induced activation of MAPK-activated protein kinases

**Figure 2:**
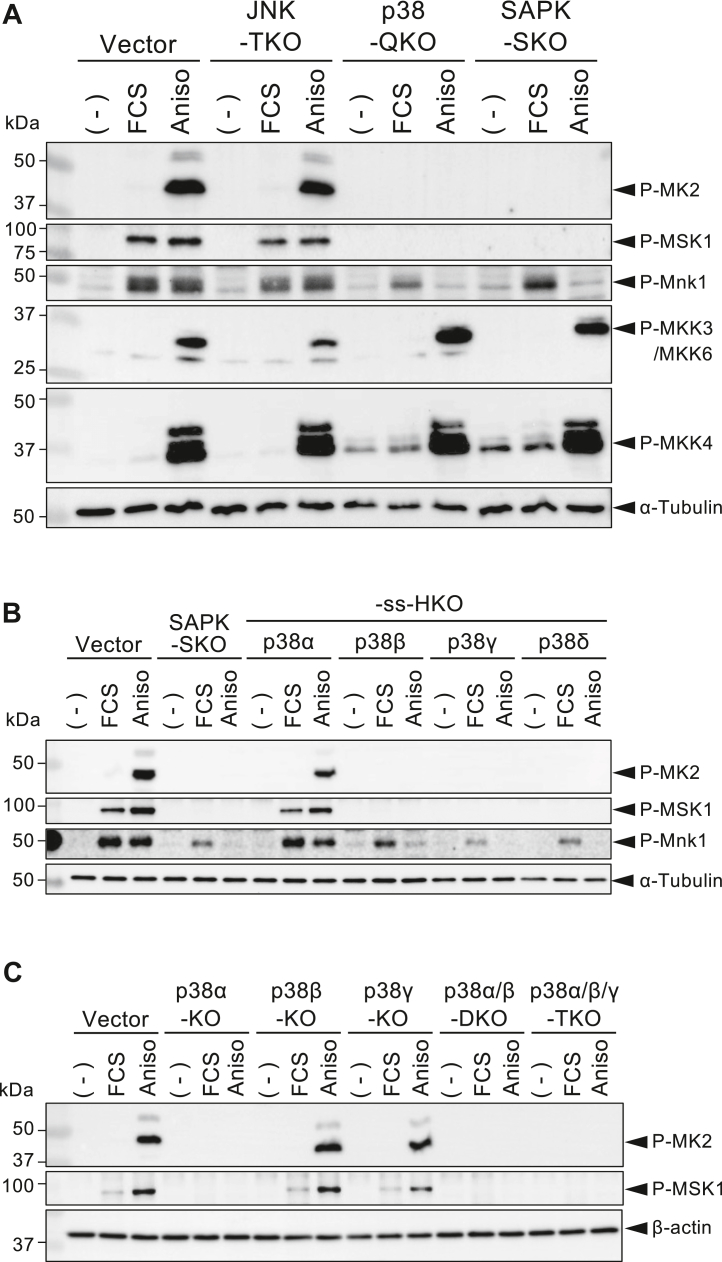
**MAPKAP kinase activation by p38 but not JNKs.** Vector control and various KO cells were serum-starved for 24 h and then unstimulated (−) or stimulated with FCS or anisomycin (Aniso) as described in the *Experimental procedures* section. Total cell lysates were analyzed by Western blot using phospho-specific antibodies. *A*, cell lysates from the vector control, JNK-TKO, p38-QKO, and SAPK-SKO cells were analyzed by Western blotting using phospho-specific antibodies for MK2(Thr334), MSK1(Thr581), Mnk1(Thr209/Thr214), MKK3(Ser189)/MKK6(Ser207), and MKK4(Ser257). A blot with anti-α-tubulin was shown as a loading control. *B*, cell lysates from the vector control, SAPK-SKO, and four types of p38-ss-HKO cells were analyzed by Western blotting using phospho-specific antibodies for MK2, MSK1, and Mnk1/2. *C*, cell lysates from the vector control, p38α-KO, p38β-KO, p38γ-KO, p38α/β-DKO, and p38α/β/γ-TKO cells were analyzed by Western blotting using phospho-specific antibodies for MK2 and MSK1. A blot with anti-β-actin was shown as a loading control. DKO, double KO; FCS, fetal calf serum; HKO, hextuple KO; JNK, c-Jun N-terminal kinase; MAPKAPK, MAPK-activated protein kinase; MK2, MAPKAPK2; QKO, quadruple KO; SAPK, stress-activated protein kinase; SKO, septuple KO; ss, sole survivor; TKO, triple KO.

MAPKs regulate cellular function through substrate phosphorylation, including the MAPK-activated protein kinase (MAPKAPK) family (21, 22), transcription factors such as the Jun/ATF family (2, 5) and the ternary complex factor (TCF) family members (23, 24, 25). To elucidate the SAPK members responsible for phosphorylation of MAPKAPK members, the FCS- or anisomycin-stimulated cells were analyzed for phosphorylation of MAPKAPK2 (MK2), MSK1, and Mnk1. As shown in Figure 2*A*, anisomycin treatment strongly induced phosphorylation of these proteins in the vector control cells, whereas FCS induced phosphorylation of MSK1 and Mnk1 but not of MK2. Anisomycin-induced phosphorylation of these kinases was similarly observed in the JNK-TKO cells but was completely absent in p38-QKO and SAPK-SKO cells. On the other hand, FCS-induced phosphorylation of Mnk1 was only partially diminished in p38-QKO and SAPK-SKO cells. These results reconfirm that p38s, but not JNKs, mediate stress-dependent activation of MKs, MSKs, and Mnks and that p38s as well as ERKs mediate FCS-dependent activation of Mnk1 (21, 22). In comparison with the control cells, basal or induced phosphorylation of MKK3 and MKK4, the upstream MAPK kinases for p38 and JNK, respectively, was enhanced in p38-QKO and SAPK-SKO cells. These results suggest a feedback pathway induced by the absence of p38 members. Analyses of the four p38-ss-HKO cell lines showed that p38α is essential for MK2 and MSK1 phosphorylation, whereas p38β and p38γ do not compensate for the absence of p38α. In contrast, Mnk1 underwent phosphorylation by both p38α and p38β (Fig. 2*B*). To determine whether functional redundancy exists among p38 family members with respect to the MAPKAPK activation, we examined individual p38 single KO cells. As shown in Figure 2*C*, anisomycin-induced phosphorylation of MK2 and MSK1 was observed equally in the vector control, p38β-KO, and p38γ-KO cells but completely disappeared in p38α-KO, p38α/β-DKO, and p38α/β/γ-TKO cells, indicating that neither p38β nor p38γ compensates for the critical function of p38α.

### Distinct contributions of SAPK members for phosphorylation of transcription factors, c-Jun, ATF2, Elk1, and CREB/ATF1

**Figure 3:**
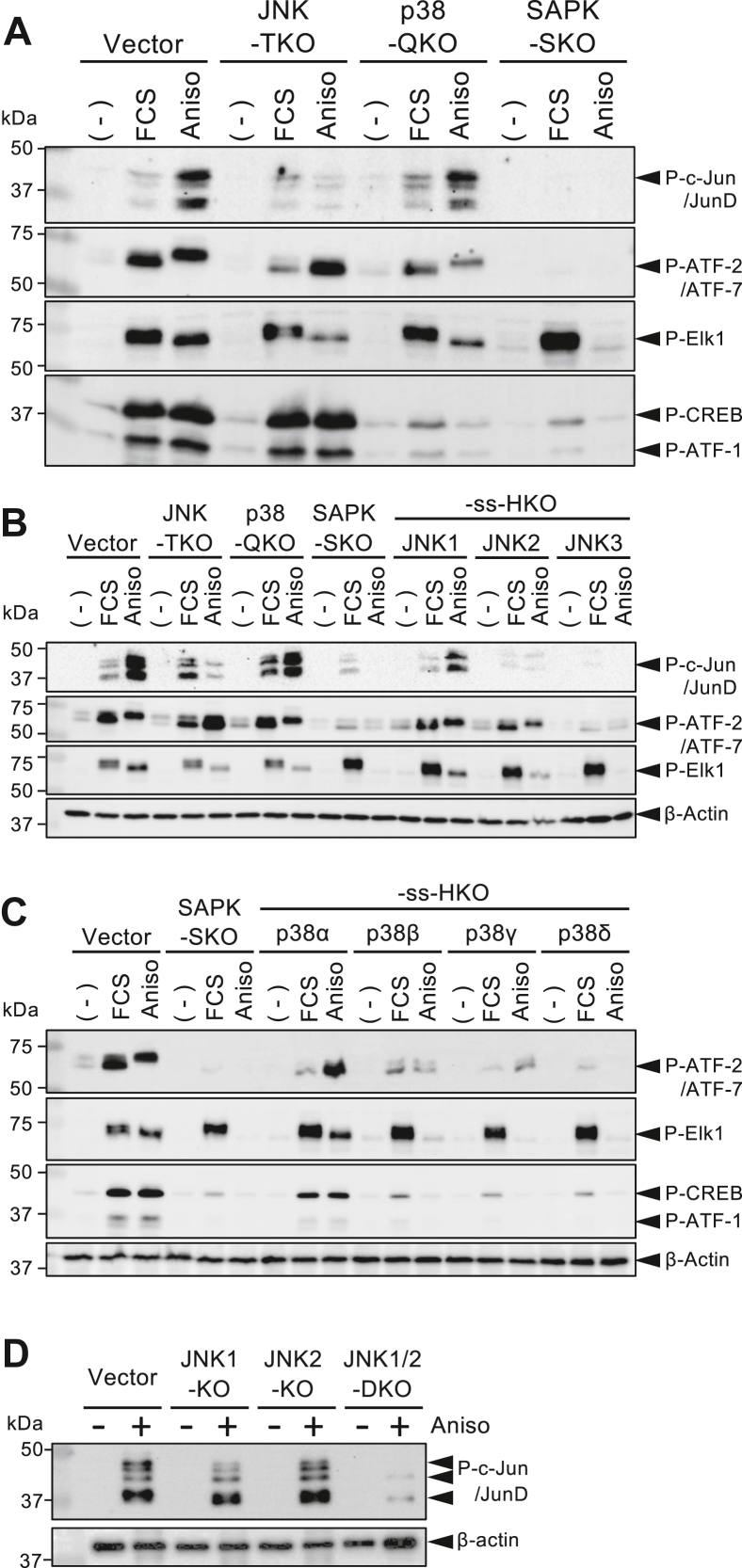
**Differential phosphorylation of c-Jun, ATF2/7, Elk1, and CREB in various SAPK-KO cells.** Total cell lysates, prepared as in Figure 2, were analyzed by Western blotting using phospho-specific antibodies. *A*, cell lysates from the vector control, JNK-TKO, p38-QKO, and SAPK-SKO cells were analyzed by Western blotting using phospho-specific antibodies for c-Jun(Ser73)/JunD(Ser100), ATF-2(Thr71)/ATF-7(Thr53), Elk1(Ser383), and CREB(Ser133)/ATF1(Ser63). *B* and *C*, cell lysates from the vector control, JNK-TKO, p38-QKO, SAPK-SKO, three types of JNK-ss-HKO, and four types of p38-ss-HKO cells were analyzed by Western blotting using the same phospho-specific antibodies as in *A*. *D*, vector control, JNK1-KO, JNK2-KO, and JNK1/2-DKO cells were stimulated with anisomycin as above, and their cell lysates were analyzed by Western blotting using the anti-phospho-c-Jun(Ser73)/JunD(Ser100) antibody. CREB, CRE-binding protein; DKO, double KO; HKO, hextuple KO; JNK, c-Jun N-terminal kinase; QKO, quadruple KO; SAPK, stress-activated protein kinase; SKO, septuple KO; ss, sole survivor; TKO, triple KO.

To identify SAPK members responsible for the phosphorylation of transcription factors, such as c-Jun/JunD, ATF2, Elk1, cyclic AMP response element (CRE)–binding protein (CREB), and ATF1, the extracellular signal–stimulated KO cell lines were analyzed by Western blotting. As shown in Figure 3*A*, anisomycin-induced phosphorylation of c-Jun(Ser73)/JunD(Ser100) was drastically reduced in JNK-TKO cells and completely absent in SAPK-SKO cells, indicating that JNKs are principal kinases in c-Jun/JunD phosphorylation triggered by the stress, with minimal p38 involvement. Analysis using ss-HKO cells showed that JNK1 was the predominant contributor to c-Jun/JunD phosphorylation (Fig. 3*B*). In contrast, the stimulation-triggered phosphorylation of ATF2(Thr71)/ATF7(Thr53) was partly reduced in both JNK-TKO and p38-QKO cells and abolished in SAPK-SKO cells (Fig. 3*A*). Interestingly, FCS-induced ATF2/7 phosphorylation was mediated primarily by JNK, whereas the anisomycin-induced ATF2/7 phosphorylation was mediated by p38. Analyses using ss-HKO cell lines demonstrated that JNK1, JNK2, and p38α are predominant in ATF2/7 phosphorylation, with a lesser contribution by p38β and p38γ (Fig. 3, *B* and *C*). As JNK3 and p38δ are not originally expressed in HeLa cells, JNK3-ss-HKO and p38δ-ss-HKO cells yielded, as expected, the same results as SAPK-SKO cells. Similarly, anisomycin-induced phosphorylation of Elk1(Ser383) was mediated by JNK1/2 and p38α, but FCS-induced Elk1 phosphorylation was enhanced in SAPK-SKO cells, suggesting ERK1/2 involvement in this phosphorylation. (Fig. 3, *A*–*C*). Also examined was the phosphorylation of CREB and ATF1, known to occur *via* RSK and/or MSK. FCS- or anisomycin-induced phosphorylation of CREB(S133) and ATF1(S63) was significantly reduced in p38-QKO and SAPK-SKO cells but not in JNK-TKO cells, indicating the exclusive contribution of p38s, especially p38α, to this (Fig. 3, *A* and *C*). To confirm the predominance of JNK1 over JNK2 for the c-Jun/JunD phosphorylation, individual JNK-single KO cells were examined. As shown in Figure 3*D*, the anisomycin-induced c-Jun/JunD phosphorylation was comparably observed in the vector control and JNK2-KO cells, slightly reduced in JNK1-KO cells, and disappeared in JNK1/2-DKO cells, indicating that JNK1 plays a major role, but JNK2 can partly compensate for the absence of JNK1, at least in c-Jun/JunD phosphorylation.

### Roles of SAPK members in activating transcription factors, AP1 and SRF/TCF, by environmental stresses

**Figure 4:**
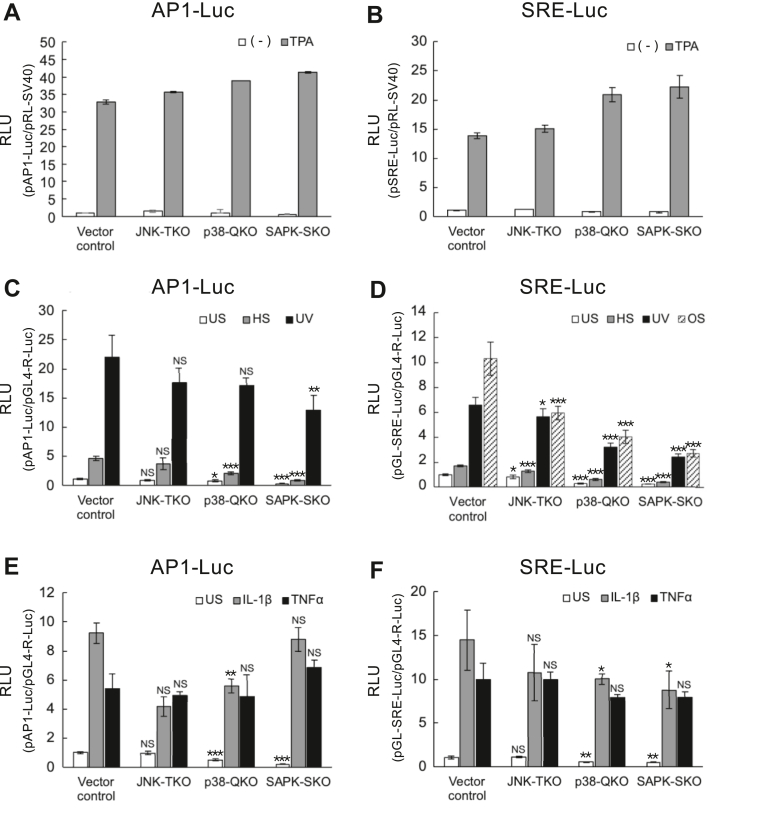
**Activation of AP-1 and SRE reporter expression in SAPK-KO cells upon growth, stress, or cytokine stimulation.** The vector control and SAPK-MKO cells were transfected with the firefly reporter plasmid, pAP1-Luc, pSRE-Luc, or pGL-SRE-Luc, together with the *Renilla* luciferase plasmid, pRL-SV40 or pGL4-Rluc. Cells were serum-starved (−) for 18 h followed by stimulation with TPA, or grown in GM (unstimulated [US]) for 18 h followed by stimulation with HS, UV, OS, IL-1β, or TNFα. Six hours after stimulation, cells were lysed, and firefly and *Renilla* luciferase activities were measured as described in the *Experimental procedures* section. In all experiments, the mean value of the US vector control cells was set at 1.0. *A* and *B*, data are presented as the mean values (n = 2). *C*–*F*, data are presented as mean ± SD (n = 3–4). Statistical analysis was performed using Student’s *t* test. *Asterisks* indicate significant difference compared with the corresponding vector control (∗*p* < 0.05; ∗∗*p* < 0.01; and ∗∗∗*p* < 0.001). AP-1, activator protein 1; GM, growth medium; HS, heat shock; IL-1β, interleukin 1; MKO, multiple KO; NS, not significant; OS, overall survival; SAPK, stress-activated protein kinase; SRE, serum response element; TNFα, tumor necrosis factor α; TPA, 12-*O*-tetradecanoylphorbol 13-acetate.

As c-Jun is an activator protein 1 (AP-1) subunit binding to the 12-*O*-tetradecanoylphorbol 13-acetate (TPA)–responsive element, and Elk1 is a member of the TCF family proteins binding to the serum response element (SRE) with their partner protein SRF (25, 26), we next examined which SAPK members are responsible for activating their respective enhancer-reporter genes in response to various extracellular stimuli. In comparison with the control cells, JNK-TKO, p38-QKO, and SAPK-SKO cells underwent slightly increased AP1- and SRE-reporter activation after TPA stimulation, suggesting the principal involvement of the ERK signaling pathway (Fig. 4, *A* and *B*). Therefore, we next tested environmental stresses, such as HS, UV-C irradiation, and osmotic shock (OS), as well as two inflammatory cytokines, interleukin-1β (IL-1β) and tumor necrosis factor α (TNFα), as these stimuli induce distinct phosphorylation and activation patterns for ERK, JNK, and p38 in HeLa cells (Fig. S3). HS- or UV-induced AP1-reporter expression underwent a slight reduction in JNK-TKO and p38-QKO cells and a greater reduction in SAPK-SKO cells (Fig. 4*C*). Similarly, HS-, UV-, or OS-induced SRE-reporter expression was significantly reduced in JNK-TKO and p38-QKO cells, with a greater reduction in SAPK-SKO cells (Fig. 4*D*). On the other hand, cytokine-induced AP1- or SRE-reporter expression was only slightly affected by the SAPK deficiency, suggesting different signaling pathways (Fig. 4, *E* and *F*). As these results suggest that SAPK deficiency affects the induced expression of endogenous genes by various environmental stimuli, we then focused on the expression of the immediate-early genes in the SAPK-KO cells.

### SAPK members differentially contribute to the induction of immediate-early genes in response to inflammatory cytokines and environmental stresses

**Figure 5:**
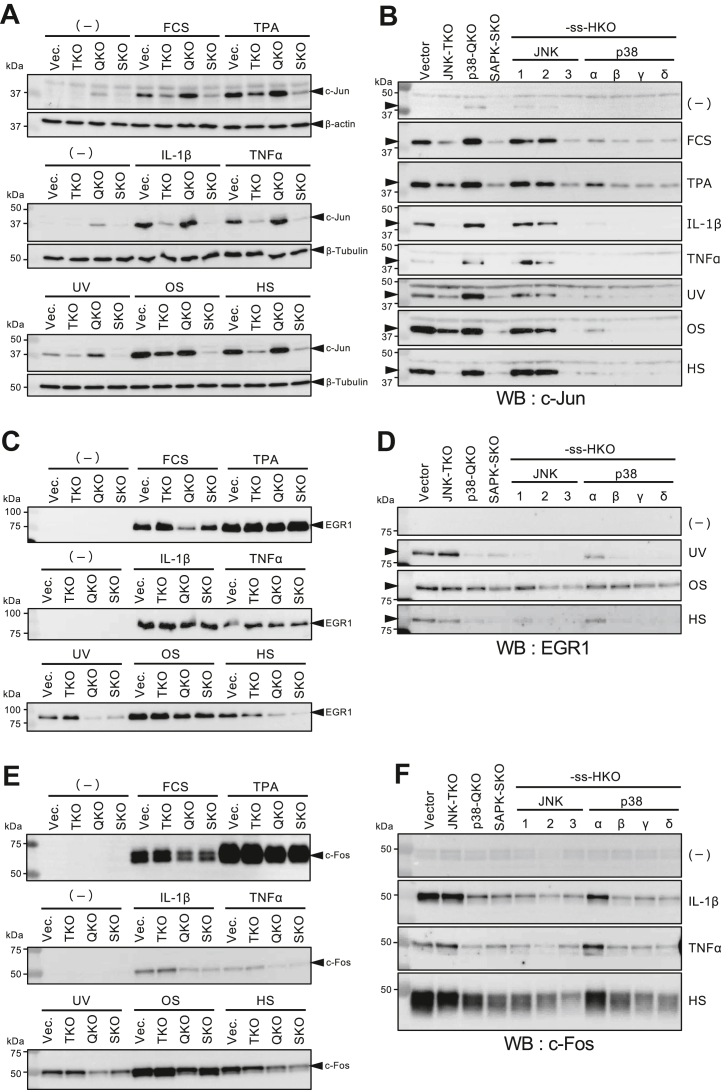
**Induced expression of c-Jun, EGR1, and c-Fos genes by various stimuli in SAPK-MKO cells.** Vector control and various KO cells were serum-starved for 24 h then unstimulated (−) or stimulated with FCS, TPA, IL-1β, TNFα, UV, OS, or HS for 120 min as described in the *Experimental procedures* section. Total cell lysates were analyzed by Western blot using the specific antibodies. *A* and *B*, expression of c-Jun in the variously stimulated vector control, JNK-TKO, p38-QKO, SAPK-SKO, and seven ss-HKO cells. *C* and *D*, expression of EGR1 in the same cells as in *A* and *B*. *E* and *F*, expression of c-Fos in the same cells as in A and *B*. FCS, fetal calf serum; HKO, hextuple KO; HS, heat shock; IL-1β, interleukin 1β; JNK, c-Jun N-terminal kinase; MKO, multiple KO; OS, overall survival; QKO, quadruple KO; SAPK, stress-activated protein kinase; SKO, septuple KO; ss, sole survivor; TKO, triple KO; TNFα, tumor necrosis factor α; TPA, 12-*O*-tetradecanoylphorbol 13-acetate.

Finally, we examined which SAPKs mediate the inducible expression of three typical immediate-early genes, c-Jun, EGR1, and c-Fos, upon various growth, inflammatory, or stress stimuli. As shown in Figure 5*A*, expression of the c-Jun gene induced by IL-1β, TNFα, or HS was mostly abolished in JNK-TKO cells and SAPK-SKO cells, indicating the essential role of JNKs. On the other hand, FCS-, TPA-, UV-, or OS-induced c-Jun expression was likely mediated not only by JNKs but also by p38s. Analyses using the ss-HKO cell lines showed that JNK1 and JNK2 contribute equally to c-Jun induction, with p38α primary among the p38 family members (Fig. 5*B*). Interestingly, c-Jun expression upon stimulation by IL-1β, TNFα, or UV in p38-QKO cells was greater than that in the control cells. Also, basal expression of c-Jun was observed even in unstimulated p38-QKO cells. The fact that c-Jun expression was lower in SAPK-SKO cells than in JNK-TKO cells would be explained by postulating two distinct functions of p38: weak Jun-phosphorylating activity and JNK-suppressing activity *via* crosstalk to the JNK’s upstream pathway. These results suggest a compensatory activation mechanism of the JNK pathway through crosstalk feedback caused by the absence of p38. This is consistent with the observation of MKK3^−/−^MKK6^−/−^ mouse embryonic fibroblasts, in which TNFα-induced c-Jun expression was increased (27). On the other hand, EGR1 gene expression induced by UV or HS was largely p38α dependent, with only a marginal contribution by JNKs, whereas the OS-induced EGR1 expression was mediated by both JNK and p38 (Fig. 5, *C* and *D*). EGR1 induction by FCS, TPA, and inflammatory cytokines was essentially unaffected in SAPK-SKO cells, suggesting that ERK or other signaling pathways are mainly involved. In contrast to c-Jun and EGR1, the cytokine-induced c-Fos expression was primarily mediated by p38α, which also, to some extent, contributed to the FCS-, UV-, OS-, or HS-induced c-Fos expression (Fig. 5, *E* and *F*). The major role of p38α for UV- or HS-induced EGR expression and for IL-1β-, TNFα-, or HS-induced c-Fos expression was confirmed by an analysis using p38 single KO cells (Fig. S4), where expression of EGR1 or c-Fos induced by these stimuli was significantly reduced in p38α-KO, p38α/β-DKO, and p38α/β/γ-TKO cells but not in p38β-KO and p38γ-KO cells compared with the vector control cells. Similar expression analyses of the JunB, JunD, FosB, Fra1, and Fra2 genes revealed various contributions by SAPKs to cytokine- or stress-induced expression of Jun/Fos family members (Fig. S5). Together, these results demonstrate the differential roles of SAPK members in the signaling pathways regulating transcription factors acting on the immediate-early genes.

## Discussion

Here, a comprehensive set of SAPK-deficient HeLa cells was established to elucidate isotype-specific or overlapping functions of individual SAPK family members. This approach was enabled by the simple and efficient method of simultaneous multigene KO described previously, with which any combination of multigene KOs may be attained by cotransfecting individual sgRNA-expressing plasmids without constructing new multicassette-assembled plasmids (20).

All SAPK-MKO cell lines proliferated with growth rates indistinguishable from that of wild-type HeLa cells, indicating that none of the SAPK members are essential for HeLa cell survival and growth. In parallel to the generation of SAPK-KO cells, we attempted to generate ERK1/2-DKO cells by the same strategy. Although ERK1- or ERK2-single KO cells were easily obtained with high efficiencies, no ERK1/2-DKO cell line was successfully generated as polyclonal populations or clones, suggesting that either ERK1 or ERK2 is essential for HeLa cell survival and/or proliferation (unpublished observation).

Previously, isotype-specific or overlapping functions of individual SAPK family members in living organisms were investigated by generating DKO mice by crossing the individual single KO mice (2, 5, 19). JNK1-, JNK2-, or JNK3-single KO mice are viable, though they exhibit impairment in T-cell differentiation or neuronal apoptosis. In contrast, JNK1/2-DKO mice exhibit embryonic lethality because of dysregulation of apoptosis in the brain, indicating overlapping functions between JNK1 and JNK2 that are not compensated by JNK3 (6, 8, 10, 11). JNK3 plays a brain-specific function, particularly in neuronal apoptosis within the hippocampus (9). On the other hand, p38α-KO mice exhibit embryonic lethality, with defects in erythroid and placental development, but p38β-, p38γ-, p38δ-KO, and p38γ/δ-DKO mice showed no apparent phenotype, suggesting that p38α is the major contributor among the four p38 members (14, 15, 16, 17, 18).

In the present study, we compared various ss-HKO cells with their parental cells and whole-member–disrupted counterparts to elucidate the relative contributions of individual SAPK members to cellular responses. Previous studies reported that MAPKAPK family members were phosphorylated by ERK and/or p38 but not by JNK (21, 22). This was confirmed in our study, along with demonstrating the phosphorylation of MK2 and MSK1 by p38α but not p38β or p38γ, and Mnk1 phosphorylation by both p38α and p38β. Also, the absence of p38 MAPK was shown to cause feedback activation of MKK3 and MKK4, as previously described (Fig. 2) (3, 4). Finally, we confirmed the previously established concept that c-Jun(Ser73)/JunD(Ser100) is phosphorylated almost exclusively by JNK, whereas ATF2(Thr71) is phosphorylated by both JNK and p38, while Elk1(S383) is phosphorylated by any of the three MAPKs. Although little contribution of p38γ was observed throughout this study, this isotype appears to participate in stress-induced ATF2 phosphorylation (Fig. 3*C*).

MAPK signaling pathways regulate immediate-early gene expression by phosphorylating and thus activating members of the proto-oncogenic transcription factors, c-Myc, c-Jun, and c-Fos, and other transcription factors, such as CREB, ATF, SRF, and TCF (28, 29). Furthermore, some Jun/Fos members are themselves immediate-early genes. We demonstrated that JNKs are the predominant contributors to c-Jun induction triggered by FCS, TPA, UV, or OS and are almost essential for IL-1β-, TNFα-, or HS-triggered c-Jun induction (Fig. 5, *A* and *B*). The c-Jun promoter region contains multiple *cis*-elements, including the Sp1, AP-1, and C/EBP sites, of which the AP-1-binding site, TPA-responsive element, was shown to be critical for c-Jun expression induced by UV and TNFα (30, 31). Previous studies showed that c-Jun expression is regulated through a positive feedback loop (32) and that the Ser63/Ser73-phosphorylated c-Jun interacts with the TCF4 coactivator to activate target genes, whereas unphosphorylated c-Jun recruits the MBD3/NuRD corepressor complex to mediate gene repression (33, 34, 35). The importance of Ser63/Ser73 phosphorylation in stress-induced apoptosis is also known (36, 37). In addition to these reports, the present study demonstrated that the phosphorylation of c-Jun at Ser63/Ser73 by JNK is a critical juncture between activation and repression of Jun’s target genes, including the c-Jun gene itself.

In contrast to c-Jun, the stress-induced expression of EGR1 depends largely on p38, especially p38α, which was almost essential for EGR1 expression triggered by UV and HS. On the other hand, both p38 and JNK partially contribute to OS-induced EGR1 expression, with a signaling pathway other than the SAPK pathway likely responsible for IL-1β- or TNFα-induced EGR1 expression (Fig. 5, *C* and *D*). The EGR1 promoter region contains a CRE, two Sp1 sites, and five SREs (38). As the SRF–TCF complex, known to bind SRE, is activated by MAPK-mediated phosphorylation of Elk1 and/or Elk4 (also called Sap1a) (24), which then interacts with the MED23 mediator protein (39), the p38-catalyzed phosphorylation of Elk1(Ser383) and/or Elk4(Ser381) is likely significant for stress-induced EGR1 expression. In addition, stress-induced CREB(Ser133) phosphorylation may contribute to EGR1 expression, as anisomycin-induced CREB phosphorylation is p38 dependent, likely *via* MSK (Figs. 2 and 3).

With regard to c-Fos expression, the cytokine-induced expression of c-Fos, whose induction magnitude was not high compared with the growth stimuli, was clearly dependent on p38α. As the importance of SRE in the inducible expression of the c-*fos* gene is extensively established (23, 25, 40), c-Fos induction triggered by FCS or TPA is likely mediated by the ERK–Elk pathway, and induction by inflammatory cytokines is mainly mediated by the p38–Elk pathway.

We recently generated the same set of SAPK-MKO cells using the monocytic leukemia cell line THP1. Analyses of FCS- or anisomycin-induced phosphorylation of MK2, ATF2/7, and CREB/ATF1 in THP1-derived MKO cells showed largely similar results to HeLa-derived MKO cells, indicating that the findings in this study are reproducible at least in this hematopoietic cell line. As a clear difference, however, we observed constitutive and a much higher level of stimuli-induced phosphorylation of JNK1/2 in p38-QKO THP1 cells, which in turn resulted in high levels of c-Jun/JunD phosphorylation and c-Jun expression (unpublished observation). This result suggests that HeLa and THP1 cells have different mechanisms for feedback crosstalk between the JNK and p38 pathways.

In conclusion, our study presents the first comprehensive KO analysis of individual SAPK members evaluating extracellular stimuli-triggered phosphorylation of signaling molecules and the induced expression of immediate-early genes. In particular, the ss-KO strategy was essential to clarify individual member-specific functions. Although we did not find p38β- or p38γ-specific functions independent of p38α, applying the ss-HKO cells derived from HeLa or other cell lines to the phosphoproteomic analysis may provide further information about their specific targets.

## Experimental procedures

### Plasmids

Targeting plasmids for the Cas9/sgRNA-mediated KO of genes for JNK1/2/3 and p38α/β/γ/δ were constructed by inserting each 21-base pair target sequence into the pX330-U6-Chimeric_BB-CBh-hSpCas9 plasmid (Addgene ID = 42230) (41). Target sequences listed in Table S1 were chosen from the target entries of the GeCKOv2 human libraries A and B (42, 43) by referring to the rankings offered by the CHOPCHOP web tool (https://chopchop.cbu.uib.no) (44).

### Cell culture and transfection

HeLa cells and derivatives were cultured in a growth medium of Dulbecco's modified Eagle's medium (Nacalai Tesque) supplemented with 5% FCS (Biosera), 100 U/ml penicillin G (FujiFilm Wako), and 100 μg/ml streptomycin (FujiFilm Wako). Transfection of HeLa cells with plasmids and subsequent puromycin selection were performed as described (20). In brief, 1 × 10^6^ cells seeded in 100-mm dishes were cotransfected with 1.5 μg of pUREF-EX (Addgene ID = 199331) (20) and 20 μg of a plasmid mixture containing an equal amount of pX330-derived plasmids (*e.g.*, 2.86 μg each of pX330-JNK1-TG1, pX330-JNK2-TG1, pX330-JNK3-TG2, pX330-p38α-TG16, pX330-p38β-TG18, pX330-p38γ-TG28, and pX330-p38δ-TG1) for generating SAPK-SKO cells. After transient selection with 10 μg/ml puromycin (InvivoGen), surviving cells were expanded and used for experiments as polyclonal cell lines.

### Stimulation and lysis of cells and Western blot analysis

Cells were seeded at 2 × 10^5^ cells/well in a 6-well plate and grown for 24 h. For the analyses of phosphorylation of signaling molecules, cells were serum-starved in Dulbecco’s modified Eagle’s medium containing 0.5% calf serum (starvation medium) for 20 h, then stimulated with FCS (25%, 20 min) or anisomycin (10 μM, 15 min; Cayman). For immediate-early gene expression experiments, cells were serum-starved for 20 h, then stimulated with FCS (25%, 120 min), TPA (500 nM, 120 min; Sigma–Aldrich), TNF-α (10 ng/ml, 120 min; PeproTech), IL-1β (10 ng/ml, 120 min; PeproTech), UV-C irradiation, OS, or HS. In the experiments of UV-C irradiation, cells in a 6-well plate were washed once with the PBS, exposed to UV-C light at 40 J/m^2^ using a Stratalinker 2400 (Stratagene), then cultured in the starvation medium for 120 min at 37 °C. For the OS, serum-starved cells were incubated for 30 min at 37 °C in the starvation medium supplemented with an additional 0.2 M NaCl and then cultured in the normal starvation medium for 90 min at 37 °C. For the HS, serum-starved cells were incubated for 30 min at 43 °C and then incubated for 90 min at 37 °C. After stimulation, cells were washed with ice-cold PBS, lysed with Laemmli's SDS sample buffer, and incubated at 85 °C for 30 min followed by 95 °C for 5 min to shear genomic DNA. Cell lysates were resolved by SDS-polyacrylamide gel electrophoresis and analyzed by Western blotting with Clarity Western ECL Substrate (Bio-Rad) using the primary antibodies listed in Table S2 and secondary antibodies, such as horseradish-peroxidase–conjugated anti-rabbit and anti-mouse antibodies purchased from DAKO.

### Luciferase reporter assay

Cells were seeded in 24-well plates at 1.0 × 10^5^ cells/0.5 ml growth medium/well and incubated for 6 h at 37 °C. Then, 0.1 ml of Opti-MEM (Gibco/ThermoFisher) containing 2 μg of the firefly luciferase reporter plasmid, pGL-SRE-Luc (formally pGL4.33-*luc2P*/SRE/Hygro; Promega E1340), pSRE-Luc (Stratagene; #219080), or pAP1-Luc (Stratagene; #219074), 0.02 μg of the *Renilla* luciferase plasmid, pRL-SV40-Luc (formally pGL4.73-*hRluc*/SV40; Promega; E6911) or pGL-Rluc (formally pGL4.70-*hRluc*; Promega; E6881), and 4 μg of polyethyleneimine (PEI-Max; Polysciences) was added to each well (*n* = 2–4). Eighteen hours after transfection, cells were fed with fresh growth medium, further incubated for 24 h, then stimulated with TPA, IL-1β, TNFα, HS, UV, or OS as described above. Six hours after stimulation, cells were lysed, with firefly and *Renilla* luciferase activities assayed by the Dual-Luciferase Reporter Assay System (Promega; E1910). Relative luminescence activities of firefly luciferase were standardized by dividing by the respective *Renilla* luciferase activities.

## Data availability

All data are contained within the article and supporting information.

## Supporting information

This article contains supporting information.

## Conflict of interest

The authors declare that they have no conflicts of interest with the contents of this article.

## Supporting information

Supporting Figuers and Tables
