# Supplementary material for: Dissecting stress-activated protein kinase (SAPK)–signaling pathways using multiplex gene knockout HeLa cells
Source: J Biol Chem. 2025 Nov 4;301(12):110901. doi: 10.1016/j.jbc.2025.110901 (PMC12704300; doi:10.1016/j.jbc.2025.110901)
Supplement: Supporting Figuers and Tables [file mmc1.pdf]

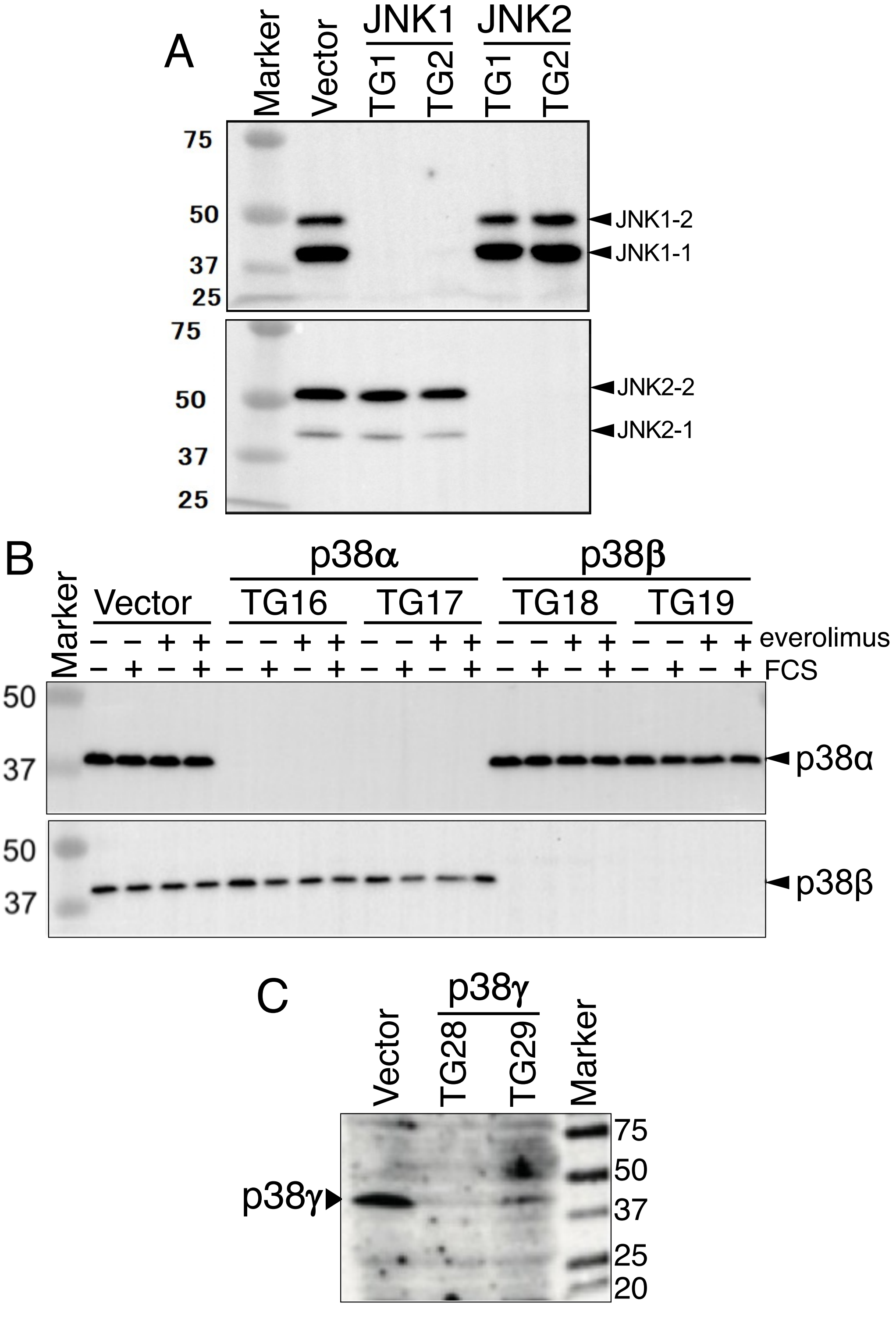

Supporting Figure S1: Estimation of knockout efficiencies of the two sgRNA constructs for each target genes.

Western blot of total cell lysates prepared from knockout cell lines generated by transfection of HeLa cells with co-transfection of a single pX330-derived targeting plasmid listed in Table S1, together with the pUREF-EX plasmid. Antibodies listed in Table S2 were used to detect JNK1 and JNK2 (A), p38α and p38β (B), and p38γ (C). In panel B, cell lysates prepared from cells treated with everolimus and/or FCS were used for another purpose, and the drug treatment is not relevant to the interpretation of this result. Control cells transfected with the pX330 empty vector (Vector) expressed JNK1, JNK2, p38α, p38β, and p38γ but not JNK3 or p38δ. In contrast, no expression of the protein products of the individual target genes was detected in any cell lines except for the pX330-p38γ-TG29-transfected cells, indicating efficient knockout of the tested genes.

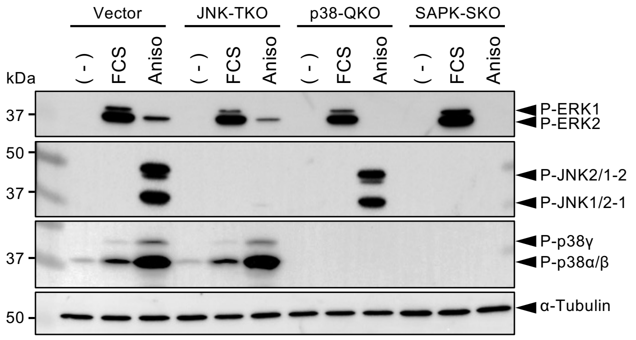

Supporting Figure S2: Stimuli-triggered activation of JNK or p38 was not affected by the disruption of the other SAPK subfamily. The vector control, JNK-TKO, p38-QKO, or SAPK-SKO cells were stimulated with FCS for 20 min or anisomycin for 15 min as described in the Experimental procedures. Total lysates were analyzed by western blot using phospho-specific antibodies for ERK1/2(T202/Y204), JNK(T183/Y185), and p38(T180/Y182).

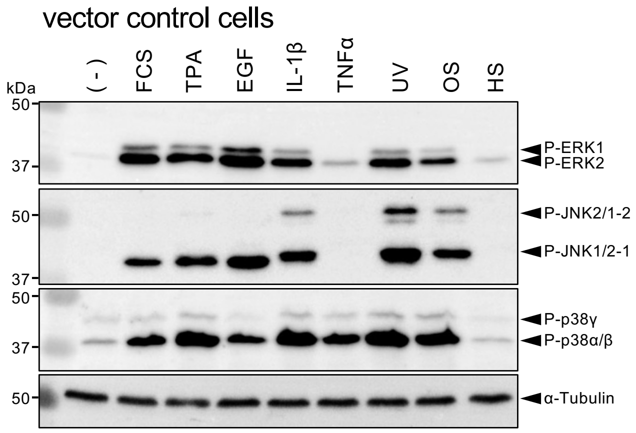

Supporting Figure S3: Growth, stress, or cytokine stimulation differentially activates three MAPK subfamilies.
The vector control cells were treated with various environmental stimuli for 15-30 min as described in Experimental procedures, and total cell lysates analyzed by western blotting using phospho-specific antibodies for ERK(T202/Y204), JNK(T183/Y185), and p38(T180/Y182).

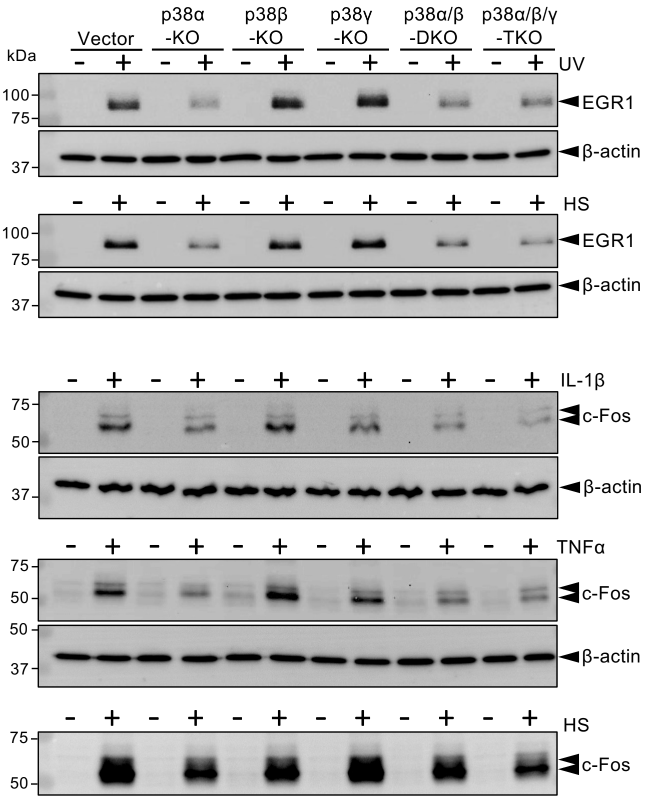

Supporting Figure S4: Induced expression of EGR1 and c-Fos genes in stress-stimulated p38-MKO cells.

Vector control, p38α-KO, p38β-KO, p38γ-KO, p38α/β-DKO, and p38α/β/γ-TKO cells were serum-starved for 24 h, and then unstimulated (-) or stimulated with UV, HS, IL-1β, and TNFα, for 120 min as in Fig. 5. Total cell lysates were analyzed by western blot using the specific antibodies for EGR1 and c-Fos.

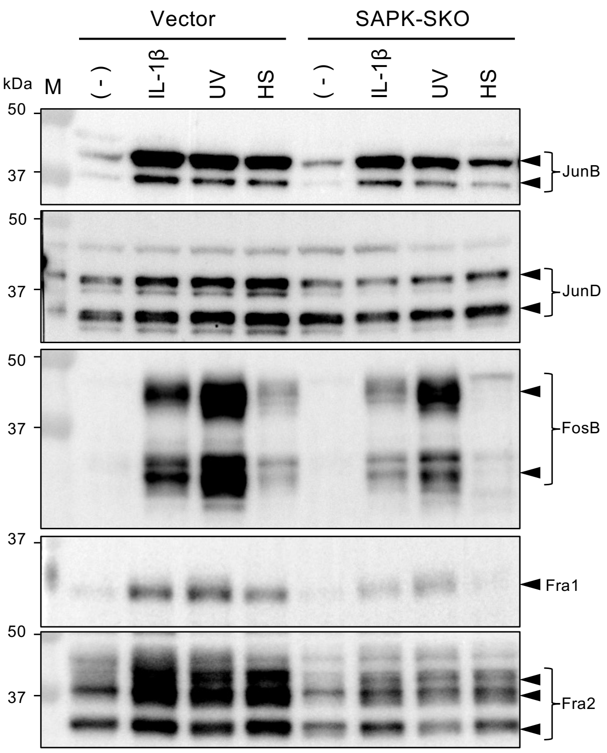

Supporting Figure S5:. Induced expression of JunB, JunD, FosB, Fra1, and Fra2 genes in SAPK-SKO cells. Vector control and SAPK-SKO cells were serum-starved for 24 h, then unstimulated (-) or stimulated with IL-1β, UV, or HS for 120 min as described in Experimental procedures. Total cell lysates were analyzed by western blot using the specific antibodies indicated on the right side.

Supporting Table S1. Target sequences for CRISPR/Cas9 knockout

Gene	HGNC Name	Chromosome	Name of Plasmid	Target sequence (5'->3')	PAM	Exon/Direction
JNK1	MAPK8	Chr.10q11.22	pX330-JNK1-TG1*	gTCGCTACTACAGAGCACCCG	AGG	Exon6/sense
			pX330-JNK1-TG2	gAGAATCAGACTCATGCCAAG	CGG	Exon3/sense
JNK2	MAPK9	Chr.5q35.3	pX330-JNK2-TG1*	gAATGGATGCTAACTTATGTC	AGG	Exon5/sense
			pX330-JNK2-TG2	gGCTGCATTTGATACAGTTCT	TGG	Exon3/sense
JNK3	MAPK10	Chr.4q21.3	pX330-JNK3-TG2*	gCACATGCCAAGAGAGCGTAC	CGG	Exon6/sense
p38α	MAPK14	Chr.6q21.31	pX330-p38α-TG16*	gTTATCTGTACCTTTAGACCT	CGG	Exon4/antisense
			pX330-p38α-TG17	gTCATTCACAGCTAGATTACT	AGG	Exon6/antisense
p38β	MAPK11	Chr.22q13.33	pX330-p38β-TG18*	gTCCACGCGCGCAGAACGTAC	CGG	Exon2/sense
			pX330-p38β-TG19	gCGACGAGCACGTTCAATTCC	TGG	Exon4/sense
p38γ	MAPK12	Chr.22q13.33	pX330-p38γ-TG28*	gCGTGGCGCATGTGCTTGAGC	AGG	Exon2/antisense
			pX330-p38γ-TG29	gACAAGTCCGTGAAGTCATCC	AGG	Exon3/antisense
p38δ	MAPK13	Chr.6p21.31	pX330-p38δ-TG30	gACAGCTCGGCCATCGACAAG	CGG	Exon2/sense
			pX330-p38δ-TG1*	gCATGCAGACGCCGAGATGAC	TGG	Exon7/sense

The lower-case "g" in the 5' end is an extra nucleotide added for efficient transcription.
*The asterisks indicate targeting plasmids used for generating multiplex knockout cells.

Supporting Table S2. Antibodies used for the western blotting

Antibody	Vendor	Cat. No.	Species	Clone ID
JNK1	Cell Signal. Tech.	#3708	mouse mAb	2C6
JNK2	Cell Signal. Tech.	#9258	rabbit mAb	56G8
JNK3	Cell Signal. Tech.	#2305	rabbit mAb	55A8
p38α	Cell Signal. Tech.	#9218	rabbit pAb	N.A.
p38β	Cell Signal. Tech.	#2339	rabbit mAb	C28C2
p38γ	Cell Signal. Tech.	#2307	rabbit pAb	N.A.
p38δ	Cell Signal. Tech.	#2308	rabbit mAb	10A8
c-Jun	Cell Signal. Tech.	#9165	rabbit mAb	60A8
JunB	Cell Signal. Tech.	#3753	rabbit mAb	C37F9
JunD	Cell Signal. Tech.	#5000	rabbit mAb	D17G2
c-Fos	Cell Signal. Tech.	#2250	rabbit mAb	9F6
FosB	Cell Signal. Tech.	#2251	rabbit mAb	5G4
Fra1	Cell Signal. Tech.	#5281	rabbit mAb	D80B4
Fra2	Cell Signal. Tech.	#19967	rabbit mAb	D2F1E
EGR1	Cell Signal. Tech.	#4154	rabbit mAb	44D5
Phospho-JNK(T183/Y185)	Cell Signal. Tech.	#4668	rabbit mAb	81E11
Phospho-p38(T180/Y182)	Cell Signal. Tech.	#4511	rabbit mAb	D3F9
Phospho-ERK1/2(T202/Y204)	Cell Signal. Tech.	#4370	rabbit mAb	D13.14.4E
Phospho-MAPKAPK2(T334)	Cell Signal. Tech.	#3007	rabbit mAb	27B7
Phospho-MSK1(T581)	Cell Signal. Tech.	#9595	rabbit pAb	N.A.
Phospho-Mnk1(T209/T214)	Cell Signal. Tech.	#2111	rabbit pAb	N.A.
Phospho-MKK3(S189)/6(S207)	Cell Signal. Tech.	#12280	rabbit mAb	D8E9
Phospho-SEK1/MKK4(S257)	Cell Signal. Tech.	#4514	rabbit mAb	C36C11
Phospho-c-Jun(S73)/JunD(S100)	Cell Signal. Tech.	#3270	rabbit mAb	D47G9
Phospho-CREB(133)/ATF1(S63)	Cell Signal. Tech.	#9198	rabbit mAb	87G3
Phospho-ATF2(T71)/ATF7(T53)	Cell Signal. Tech.	#15411	rabbit mAb	A8J7P
Phospho-Elk1(S383)	Santa Cruz Biotech.	sc-8406	mouse mAb	B-4
β-tubulin	FUJIFILM Wako	#014-25041	mouse mAb	10G10
α-tubulin(HRP-conjugated)	MBL Life Sci.	#PM054-7	rabbit pAb	N.A.
β-actin(HRP-conjugated)	MBL Life Sci.	#PM053-7	rabbit pAb	N.A.
Mouse Ig (HRP-conjugated)	Agilent Dako	P044701-2	goat pAb	N.A.
Rabbit Ig (HRP-conjugated)	Agilent Dako	P039901-2	swine pAb	N.A.
